# Genome Sequence of Antibiotic-Producing *Bacillus amyloliquefaciens* Strain KCTC 13012

**DOI:** 10.1128/genomeA.01121-15

**Published:** 2015-10-01

**Authors:** Haeyoung Jeong, Seung-Hwan Park, Soo-Keun Choi

**Affiliations:** a Super-Bacteria Research Center, Korea Research Institute of Bioscience and Biotechnology (KRIBB), Daejeon, Republic of Korea; b Biosystems and Bioengineering Program, Korea University of Science and Technology (UST), Daejeon, Republic of Korea

## Abstract

We report the 4.0-Mb draft genome sequence of *Bacillus amyloliquefaciens* (syn. *Bacillus velezensis*) KCTC 13012, which exhibits a broad spectrum of antagonistic activity against bacteria and fungi and promotes plant growth as well. The genome contains an array of biosynthetic gene clusters for secondary metabolites that are comparable to those in *Bacillus amyloliquefaciens* subsp. *plantarum* FZB42^T^.

## GENOME ANNOUNCEMENT

Genera in the Bacillales, including *Bacillus* and *Paenibacillus*, are known to encode a variety of biosynthetic gene clusters for secondary metabolites (1, 2). For example, 8.5% of the *Bacillus amyloliquefaciens* subsp. *plantarum* FZB42^T^ genome is devoted to the synthesis of polyketides, nonribosomal lipopeptides, or siderophores (3). The producers for these secondary metabolites are not only applicable for biocontrolling agents suppressing crop pathogens, but are also a potential source for novel antibiotics against emerging multidrug-resistant bacteria (4). KCTC 13012 (= CR-502) was initially reported as a type strain of the novel species *B. velezensis* (5); however, it was later proposed that *B. velezensis* should be considered a later heterotypic synonym of *B. amyloliquefaciens* (6). This strain was chosen for the present study because it shows a broad spectrum of antagonistic activity against Gram-positive and Gram-negative bacteria as well as plant-pathogenic fungi; in addition, it shows plant growth-promoting activity (data not shown).

KCTC 13012 was purchased from the Korean Collection for Type Cultures (Daejeon, Republic of Korea). Genome sequencing was carried out at the National Instrumentation Center for Environmental Management in Seoul National University (Seoul, Republic of Korea) using the Illumina HiSeq 2000 platform. In all, 101-nucleotide (nt) paired-end reads, totaling ~1,000× coverage, were produced from a ca. 470-bp-long genomic library. Quality trimming, filtering by length, and a *de novo* assembly were conducted using the CLC Genomics Workbench v8.0.2, resulting in 30 contigs with a 46.3% G+C content (total length of 4,039,360 bp, maximum contig length of 1,067,963 bp, and *N*_50_ of 612,006 bp). The average nucleotide identity values with close strains, as calculated by JSpecies (7) using BLASTN, were 98.3% (*B. amyloliquefaciens* subsp. *plantarum* FZB42^T^), 98.2% (*B. velezensis* G341) (8), and 93.8% (*B. amyloliquefaciens* subsp. *amyloliquefaciens* DSM 7^T^), which is in accordance with the recent study showing that the genomes of the *B, amyloliquefaciens* subsp. *plantarum* group are highly conserved (9). Automatic genome annotation conducted using the RAST server (10) identified 4,089 coding sequences and 91 RNAs, 53% of which were assigned subsystems. We were unable to find any putative plasmids from the assemblies.

With the exception of *nrsABCDEF*, KCTC 13012 has homologous gene clusters for the biosynthesis of all the other secondary metabolites present in FZB42. Because gene clusters for fengycin and surfactin biosynthesis have been found on several short contigs as fragments, the presence of these two gene clusters was confirmed through mapping sequence reads on the FZB42 reference genome, whereas the complete gene cluster structures for the others were represented on the *de novo* assemblies. A read-mapping analysis showed that KCTC 13012 lacks a prophage-like region (*yotN-yokA*) and a segment encompassing *nrsF* through RBAM_027480, which are present in FZB42. Meanwhile, a prophage-containing contig_8 (53.1 kb) represents a KCTC 13012-specific region. Our results will provide further insight into the genomic basis of the diversity of the *B. amyloliquefaciens* species and its secondary metabolite biosynthetic potential.

### Nucleotide sequence accession numbers.

The whole-genome shotgun project of KCTC 13012 was deposited at DDBJ/EMBL/GenBank under the accession number LHCC00000000. The version described in this paper is version LHCC01000000.
